# New-Onset Heart Failure From Viral Myocarditis and Inflammatory Cardiomyopathy: A Case Report

**DOI:** 10.7759/cureus.25737

**Published:** 2022-06-07

**Authors:** Bir P Singh, Kelvin Noronha, Johnathan Frunzi, Francisco A Brea Abreu

**Affiliations:** 1 Internal Medicine, HCA Florida Trinity, Trinity, USA

**Keywords:** bronchial asthma exacerbation, congestive heart failure, viral cardiomyopathy, echocardiography - heart failure - valvular heart disease, viral-induced myocarditis

## Abstract

An inflammatory cardiomyopathy is a form of nonischemic cardiomyopathy that results from myocarditis associated with cardiac dysfunction and ventricular remodeling. It can be caused by a wide array of pathogens and toxins. We present a case of a 69-year-old female with a history of asthma who was admitted to our facility with recurrent asthma exacerbations, likely triggered by viral upper respiratory tract infections. In 5 months, serial echocardiograms showed a significant decline in her left ventricular systolic and diastolic function. Cardiac catheterization showed no clinically significant coronary artery disease. Despite normal renal function, her troponin remained elevated. This is an interesting case of a viral upper respiratory tract infection that led to myocarditis and ultimately resulted in inflammatory cardiomyopathy.

## Introduction

Inflammatory cardiomyopathy is predominantly caused by viruses such as coxsackie, echovirus, and adenovirus, but it can also result from bacterial, protozoal, or fungal infections and a wide range of toxins and immune-mediated illnesses [1]. Immune-mediated and direct cytotoxic destruction of myocardial tissue plays a role in pathogenesis. Direct invasion and replication within and around the myocardium cause myocardial necrosis. Viral activation of the immune system can initiate an autoimmune response, causing cytokine-mediated cardiotoxicity, and resulting in myocardial injury. Most patients recover without further sequelae, but some patients will undergo ventricular remodeling and develop inflammatory cardiomyopathy.

Clinical manifestations vary and range from chest pain and dyspnea to cardiogenic shock and sudden cardiac death [2]. Despite significant research into immune-modulatory therapies, the treatment of patients with suspected myocarditis is mainly supportive. Severe myocarditis causing cardiogenic shock requires referral to tertiary care centers for cardiac transplant evaluation [3].

## Case presentation

**Figure 1:**
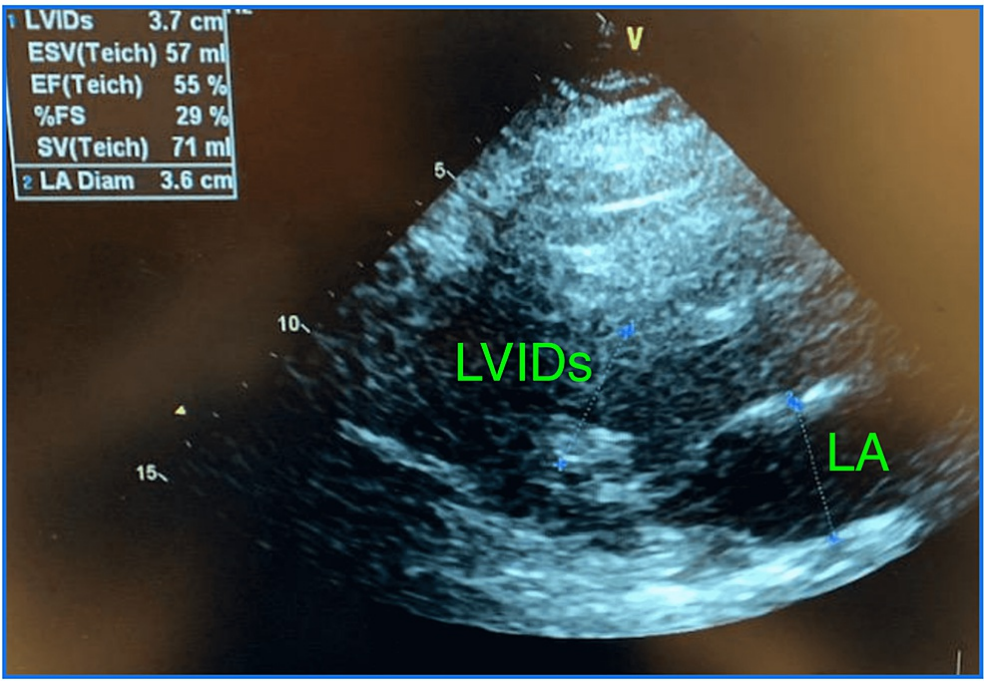
Normal left ventricular systolic function with ejection fraction of 55% Normal left ventricular internal diameter end-systole of 3.7 cm (normal 2-4 cm) on first admission for asthma exacerbation. LVIDs: left ventricular internal diameter end systole; ESV: end-systolic volume; EF: ejection fraction; SV: stroke volume; LA: left atrium; LA Diam: left atrial diameter

A 69-year-old female with a past medical history of chronic kidney disease stage III, hypertension, poorly controlled type II diabetes mellitus presented with worsening dyspnea and dry cough for one week. Her symptoms did not improve with her albuterol inhaler. She was admitted for an acute asthma exacerbation that was treated with nebulized albuterol and ipratropium, azithromycin, ceftriaxone, and steroids. An echocardiogram showed normal left ventricular systolic function with an estimated ejection fraction of 55-60%, without regional wall motion abnormalities, diastolic dysfunction, or valvular disease (Figure 1). Her symptoms improved, and she was discharged on a steroid taper.

**Figure 2:**
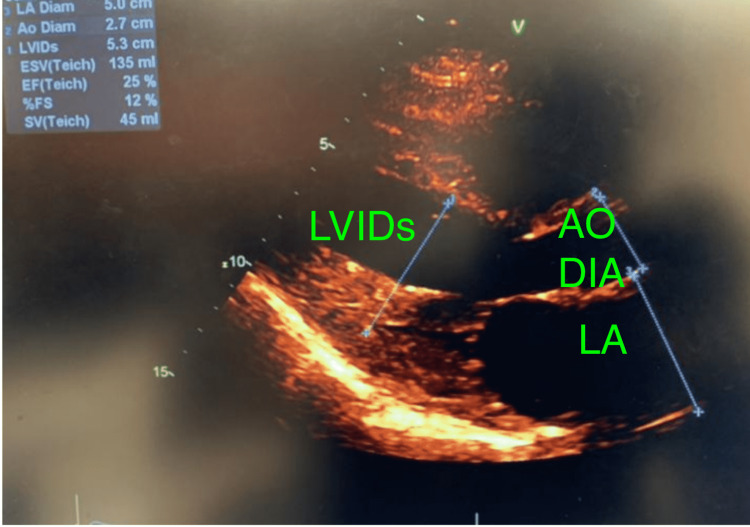
Reduced left ventricular systolic function with ejection fraction of 25% Dilated left atrium measuring 5.0 cm and increased left ventricular internal diameter end-systole of 5.3 cm (normal 2-4 cm) on hospitalization for second asthma exacerbation 5 months later. LA: left atrium; LA Diam: left atrium diameter; Ao Diam: aorta diameter; LVIDs: left ventricular internal diameter end systole; ESV: end systolic volume; EF: ejection fraction; SV: stroke volume

**Figure 3:**
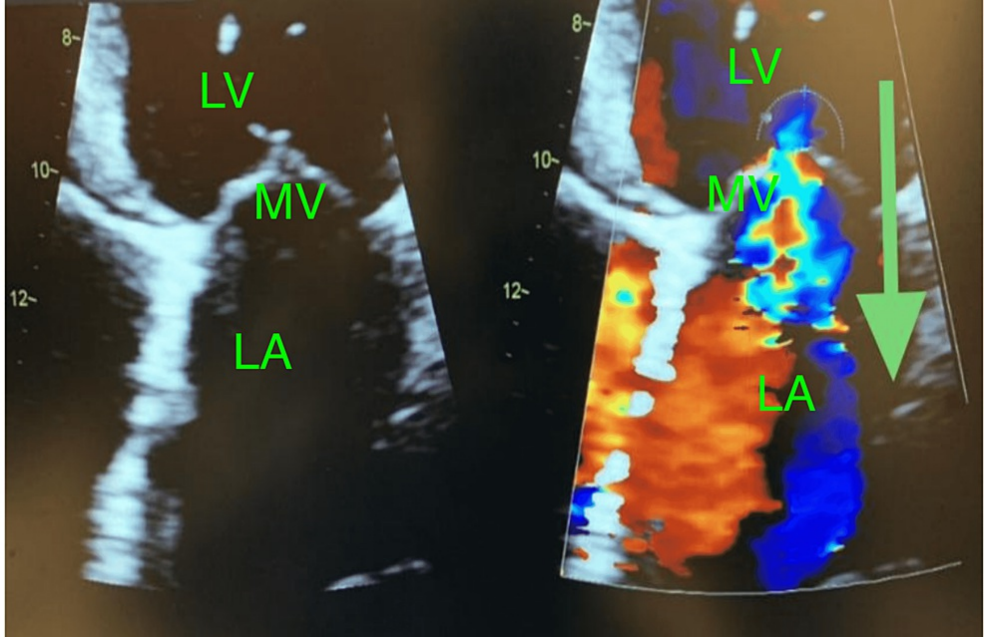
Moderate regurgitation of blood from left ventricle to left atrium across the mitral valve in the direction of the green arrow LV: left ventricle; LA: left atrium; MV: mitral valve

Five months later, this patient presented to the hospital again with an acute asthma exacerbation, requiring respiratory support with bi-level positive airway pressure and admission to the intensive care unit. Both C-reactive protein and erythrocyte sedimentation rate were elevated. Assays for coronavirus, influenza A/B, mycoplasma, chlamydia, treponema pallidum, and hepatitis B and C panels were negative. An echocardiogram during this admission showed new-onset left ventricular systolic dysfunction with an ejection fraction of 35-40%, diffuse hypokinesis, grade II diastolic dysfunction, and moderate mitral regurgitation (Figures 2-3). The patient was found to have mildly elevated troponin (0.264-0.402 ng/mL, normal < 0.015). Left heart catheterization showed no obstructive coronary artery disease.

**Figure 4:**
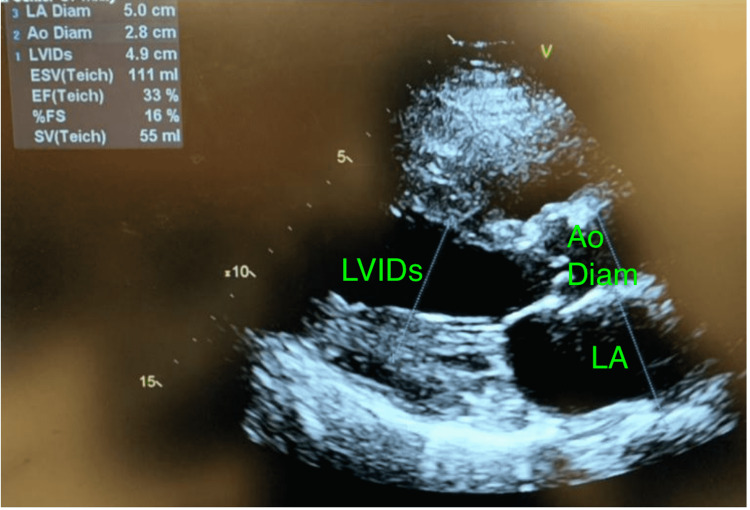
Improvement in left ventricular systolic function with ejection fraction increasing from 25 to 33% Decreased left ventricular internal diameter end systole from 5.3 to 4.9 cm after treatment with dobutamine. LVIDs: left ventricular internal diameter end systole; ESV: end-systolic volume; EF: ejection fraction; SV: stroke volume; LA: left atrium; LA Diam: left atrial diameter

Over the following months, this patient was readmitted for heart failure exacerbation mainly because of medication non-compliance. On one admission, her volume overload was refractory to intravenous diuretics. She was started on a dobutamine drip at a fixed rate with close monitoring of her electrolytes and telemetry events. Dobutamine stimulated contractility and increased renal perfusion. Within four days, she generated nearly 8 L of urine output with a slight improvement in her ejection fraction (Figure 4).

**Figure 5:**
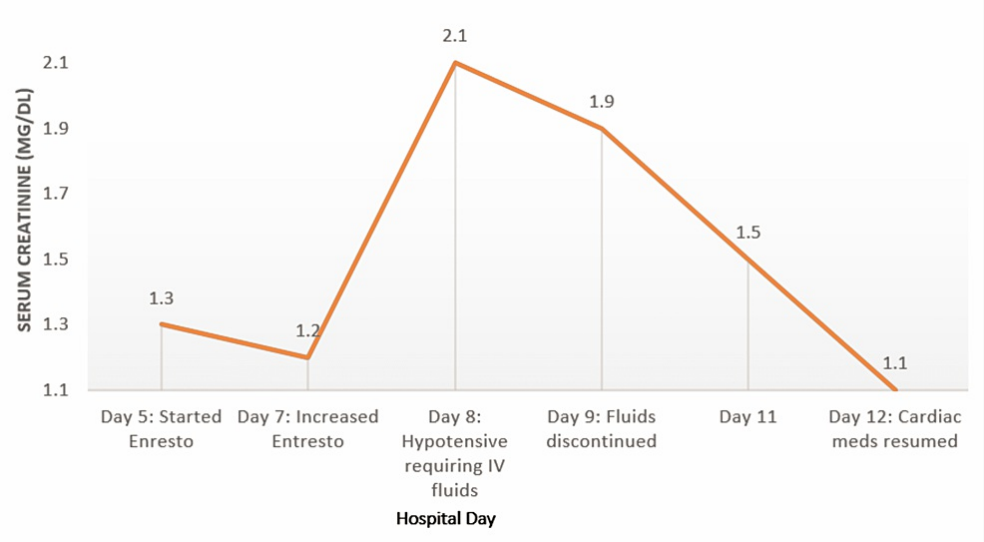
Changes in serum creatinine according to hospital day

During the hospital course, the patient was started on guideline-directed medical therapy for her cardiomyopathy with metoprolol succinate, spironolactone, and sacubitril-valsartan. Low-dose sacubitril-valsartan was started after discontinuing lisinopril and waiting 72 hours to allow for a washout to occur. The patient’s blood pressure, heart rate, and renal function remained stable. After increasing the dose, however, her renal function worsened significantly and creatinine increased by nearly 50%. She became hypotensive, requiring fluid resuscitation. Her core cardiac medications had to be held temporarily (Figure 5). As her renal function recovered, she was discharged on metoprolol succinate, spironolactone, and sacubitril-valsartan was discontinued.

## Discussion

There are multiple interesting aspects and important learning points to this case. Most interesting is the sudden decrease in the patient’s systolic function. Within 5 months, her ejection fraction dropped from 55-60% to 35-40% in the absence of coronary artery disease. We suspect that a viral infection triggered her asthma exacerbation and caused viral myocarditis, resulting in inflammatory cardiomyopathy.

Endomyocardial biopsy (EMB) is the gold standard for diagnosis because it shows histological evidence of myocardial inflammation. Its utility is limited because of sampling error from patchy inflammatory infiltrates and variability in observer interpretation [4]. According to guidelines from the American College of Cardiology and American Heart Association, EMB has few indications in evaluating patients with cardiomyopathy. Class I indications include patients with new-onset heart failure (<2 weeks) associated with normal or dilated left ventricle with hemodynamic instability. Patients with new-onset heart failure within 2 weeks to 3 months with left ventricular dilation, new ventricular arrhythmias, or a second or third-degree atrioventricular block are also class I indications, none of which our patient met [5].

Cardiovascular magnetic resonance (CMR) imaging is an alternative diagnostic modality, but findings tend to be non-specific. Since CMR was unavailable at our facility, it was not performed, but other diagnoses were excluded. There were no characteristic echocardiographic findings to suggest Takotsubo cardiomyopathy, hypertrophic obstructive cardiomyopathy, amyloidosis, or organic valvular heart disease. Furthermore, the patient had no history of congenital heart disease, hemochromatosis, no history of alcohol consumption or recreational drug use, or exposure to chemotherapy agents or other toxins.

Regardless of the etiology of cardiomyopathy, it should be managed with beta-blockers, angiotensin-converting enzyme inhibitors (ACEI), or angiotensin II receptor blockers (ARB) [6].

Based on the results of the clinical trial, PARADIGM-HF (Prospective Comparison of ARNI (Angiotensin Receptor-Neprilysin Inhibitor) with ACEI to Determine Impact on Global Mortality and Morbidity in Heart Failure), ARNI combined with ARB reduces all-cause mortality and heart failure hospitalizations when compared to ACEI alone in systolic heart failure [7]. This trial also showed that the drug had an acceptable safety profile over a median follow-up of 27 months. Despite sacubitril-valsartan being guideline-directed medical therapy in treating systolic heart failure, this case highlights that slight dose adjustments can cause severe hypotension and renal dysfunction, meaning that patients should be closely monitored for these adverse effects.

Another point is the role dobutamine can play in managing acute decompensated heart failure that is refractory to diuretics. Dobutamine’s sympathomimetic and vasodilation properties make it effective in treating volume overload refractory to diuretics. Further studies are required to investigate this use.

## Conclusions

A viral illness can precipitate myocarditis and cause inflammatory cardiomyopathy. When volume overload in systolic heart failure is refractory to diuretics, it may improve with inotropic support with dobutamine. Though guideline-directed medical therapy for treating heart failure includes sacubitril-valsartan, it can cause renal impairment, meaning that these patients require close monitoring.
